# Delayed viral vector mediated delivery of neurotrophin-3 improves skilled hindlimb function and stability after thoracic contusion

**DOI:** 10.1016/j.expneurol.2022.114278

**Published:** 2023-02

**Authors:** Jared D. Sydney-Smith, Alice M. Koltchev, Lawrence D.F. Moon, Philippa M. Warren

**Affiliations:** The Wolfson Centre for Age-Related Diseases, Guy's Campus, King's College London, London Bridge, London SE1 1UL, UK

**Keywords:** Neuroplasticity, Neurotrophin-3, Adeno-associated viral vector, Contusion, Spinal cord injury, Proprioception, H-reflex, Hyperreflexia

## Abstract

Intramuscular injection of an Adeno-associated viral vector serotype 1 (AAV1) encoding Neurotrophin-3 (NT3) into hindlimb muscles 24 h after a severe T9 spinal level contusion in rats has been shown to induce lumbar spinal neuroplasticity, partially restore locomotive function and reduce spasms during swimming. Here we investigate whether a targeted delivery of NT3 to lumbar and thoracic motor neurons 48 h following a severe contusive injury aids locomotive recovery in rats. AAV1-NT3 was injected bilaterally into the *tibialis anterior, gastrocnemius* and *rectus abdominus* muscles 48-h following trauma, persistently elevating serum levels of the neurotrophin. NT3 modestly improved trunk stability, accuracy of stepping during skilled locomotion, and alternation of the hindlimbs during swimming, but it had no effect on gross locomotor function in the open field. The number of vGlut1^+^ boutons, likely arising from proprioceptive afferents, on *gastrocnemius* α-motor neurons was increased after injury but normalised following NT3 treatment, suggestive of a mechanism in which functional benefits may be mediated through proprioceptive feedback. *Ex vivo* MRI revealed substantial loss of grey and white matter at the lesion epicentre but no effect of delayed NT3 treatment to induce neuroprotection. Lower body spasms and hyperreflexia of an intrinsic paw muscle were not reliably induced in this severe injury model suggesting a more complex anatomical or physiological cause to their induction. We have shown that delayed intramuscular AAV-NT3 treatment can promote recovery in skilled stepping and coordinated swimming, supporting a role for NT3 as a therapeutic strategy for spinal injuries potentially through modulation of somatosensory feedback.

## Highlights

• Functional recovery following delayed delivery of NT3 following thoracic contusion.
• NT3 restored animal stepping accuracy, trunk stability and synchronised movement.
• NT3 mediated recovery through normalisation of vGlut1+ boutons.
• 250kDyn thoracic contusion does not reliably result in signs of spasticity.

## 1 Introduction

Most traumatic spinal cord injuries (SCIs) are incomplete, leaving patients with some degree of residual sensory and motor function below the neurological level of the injury (National Spinal Cord Injury Statistical Centre, 2022) while causing life-altering deficits including limb paralysis and spasticity. 25–55% of SCIs occur within the thoracic or lumbar spinal cord, causing reduced lower limb function and trunk instability (Divanoglou and Levi, 2009; Pickett et al., 2003; Singh et al., 2014). This is initiated following damage to the descending supraspinal and propriospinal axons which project below the lesion and are critical for specific functions, such as fine locomotor control and synchronised movements (Jordan et al., 2008).

Some degree of spontaneous motor recovery occurs in both human patients and pre-clinical SCI models following incomplete injuries due to the formation of novel circuits which bypass the injury site to reinnervate pathways below the lesion and re-establish basic motor function (Nishimura et al., 2007; Zorner et al., 2014; Zörner et al., 2010; Tohyama et al., 2017). Proprioceptive feedback involving vGlut1+ afferents is essential for this motor recovery (Edgerton et al., 2008; Rossignol and Frigon, 2011; Takeoka et al., 2014), enabling suprathreshold signals controlling movement to reach the spinal cord circuits, stabilising, and refining motor output. Nonetheless, use of any residual function and attempts to further recovery through rehabilitation can be disrupted by the development of spasticity which occurs in up to three quarters of patients (Maynard et al., 1990; Skold et al., 1999), and results in involuntary spasms, rigidity and exaggerated reflexes termed hyperreflexia (Mukherjee and Chakravarty, 2010; Pandyan et al., 2005). As such, it is important to establish reliable strategies to limit the formation of spasticity whilst enhancing functionally effective circuit reformation from the descending axons to motoneurons within the lumbar spinal cord.

Neurotrophic factors support the activity and function of descending spared axons following SCI (Da Silva and Wang, 2011). Neurotrophin-3 (NT3) is a growth factor required for correct connectivity between proprioceptive afferents from muscle to the spinal cord (Boyce and Mendell, 2014a; Boyce and Mendell, 2014b). We, and others, have provided supplementary NT3 in animal models of neurological injury and shown that it enhances neuroplasticity and improves sensorimotor recovery in mice, rats and cats (Kakanos and Moon, 2019; Boyce and Mendell, 2014a; Boyce and Mendell, 2014b; Fortun et al., 2009; Krupka et al., 2017; Ollivier-Lanvin et al., 2015; Sydney-Smith et al., 2021a; Han et al., 2019; Duricki et al., 2021; Duricki et al., 2019). In the clinic, elevated NT3 is well-tolerated and has been shown to not cause pain when given peripherally (Parkman et al., 2003; Coulie et al., 2000; Chaudhry et al., 2000; Sahenk, 2007; Sahenk et al., 2014). In rats, delivery of NT3 via injection of AAV1 into forelimb flexor muscles reversed some sensorimotor deficits after bilateral corticospinal tract lesion in the brainstem (Kathe et al., 2016) and improved functional outcomes after mid cervical contusion injury (Sydney-Smith et al., 2021a). Similarly, a recent study assessed whether AAV1-NT3 induced therapeutic effects when injected into hindlimb muscles 24-h after a T9 thoracic contusion (Chang et al., 2019). NT3 treatment, and hindlimb rehabilitation, reduced the presence of hindlimb spasms during swimming and normalised hyperreflexia in the hind paw. There was, however, no synergistic effect of rehabilitation and NT3 treatment on functional and electrophysiological outcomes.

We hypothesised that intramuscular injection of AAV1-NT3 into muscles innervated by thoracic and lumbar spinal motor neurons would enhance skilled motor function and potentially alleviate hindlimb hyperreflexia and spasticity if delivered at a clinically relevant 48-h following the initial trauma. We show that severe thoracic contusion led to persistent hindlimb deficits but little, if any, lower body spasm, or hyper-reflexivity of an intrinsic hindpaw muscle. Nevertheless, 48 h-delayed NT3 treatment improved skilled locomotor function and hindlimb alternation during swimming potentially due to modifications of vGlut1 signalling on proprioceptive afferents.

## 2 Methods

### 2.1 Ethical approval and animal welfare

Experiments were approved by the King's College London Animal Welfare and Ethical Review Board and were conducted in accordance with UK Animals (Scientific Procedures) Act 1986 (ASPA) under Home Office Project Licence number P53631BC2. During all experiments, data processing, and analysis the investigators were blind to the treatment group of each animal. An independent third party coded and randomly assigned animals to treatment groups in an alternating pattern prior to injury without knowledge of baseline behavioural assessments and electrophysiological recordings.

Animals were housed in groups of four or five, exposed to a normal 12-h dark-light cycle at 21 °C with environmental enrichment and access to food and water ad libitum. The health and welfare of all animals was monitored daily by veterinary staff and the study investigators at King's College London and was in accordance with the Animal Welfare Act 2006. 28 female Wistar rats (256 g ± 29 g; Envigo; RccHan:WIST) were used in this study. Of the 28 animals, one died three days after contusion injury, with all other animals making uneventful recoveries. One animal was excluded from the study due to regaining near perfect locomotion in open field (BBB score of 20) within three weeks of injury. Animals were divided into three groups: 1) contusion + NT3 (NT3, *n* = 11); 2) contusion + PBS (PBS, *n* = 12); and 3) uninjured + no treatment (sham, *n* = 4). PBS was used as a control group as it was the vehicle used for treatment application (Tang et al., 2020; Johnston et al., 2021; Rghei et al., 2021; Aguilà et al., 2020; Sahenk et al., 2014). Our previous work, and that of others, has shown that intramuscular injection of AAV1-CMV-GFP into rodents causes no detectable alterations in effects on the inflammatory response, neuroinflammation, or behaviour after neurological trauma, including SCI (Petrosyan et al., 2014; Duricki et al., 2016b; Kathe et al., 2016). In adherence to the NC3Rs and ARRIVE guidelines, this additional negative control group has not been repeated in this study. Following injury, animals were housed in mixed cages comprising injured animals of both NT3 and PBS treated animals for the duration of the study. Sham animals were housed together in a separate cage.

### 2.2 Injury and treatment application

#### 2.2.1 Thoracic contusion injury

Rats were induced with Isoflurane (5%; Zoetis, UK Ltd) at 1 l.min^−1^ O_2_ flow and maintained with 2.5% isoflurane throughout surgery. Preoperatively 5 mg/kg Enrofloxacin (Baytril) and 5 mg/kg carprofen (Carprieve) were injected subcutaneously (s.c.) and a homeothermic heat pad (Harvard Apparatus) maintained body temperature at 37±1̊C. Upon reaching a surgical plane of anaesthesia, eye ointment (Viscotears) was applied and the region around the lower thoracic vertebrae was shaved and the skin sterilised.

A 2 cm dorsal midline incision from T8-T11 vertebrae was performed and the spinotrapezius muscles retracted to expose the vertebral column. A complete laminectomy of the T8 vertebra was performed and the vertebral column stabilised in the Infinite Horizon contusion impactor (Precision Systems and Instrumentation). Animals received a single bilateral contusion, of 250 kDyn force with 0 s dwell time using a 2.5 mm diameter impact tip (Precision Systems Instrumentation), at the T9 spinal segment. Muscle layers and the skin were sutured in two layers (4–0 Vicryl; Ethicon) and animals given saline (5 ml; s.c.). Sham animals received a full T8 laminectomy, without clamping in the Infinite Horizons impactor, and all pre-and post-operative drugs.

For five days after injury animals continued to receive; warm saline (s.c.), 5 mg/kg Baytril, and 5 mg/kg Carprieve. Animals were unable to void their bladders naturally for the first week after injury, necessitating manual bladder emptying three times per day. Once spontaneous voiding was recovered, bladder volume and voiding was monitored daily to ensure no infections occurred.

#### 2.2.2 Adeno-associated virus encoding neurotrophin-3

An AAV transfer plasmid, AAVspNT3, was cloned to contain the full length human prepro-neurotrophin-3 under a CMV promoter. The neurotrophin-3 coding DNA sequence (NM_002527.4), corresponding to isoform 2 precursor protein including the secretory signal, was flanked by splice donor/splice acceptor sites and terminated in a beta globin poly(A) sequence (gifted by Prof. Fred Gage, Salk Institute). This was packaged into an AAV serotype 1 (University of Pennsylvania Viral Vector Core facility; custom batch CS1232). This vector (referred to as AAV-NT3) was titered using digital droplet PCR (6.2 × 10^13^ genomic copies (gc)/ml; Viral Vector Core) and in our lab via qPCR (1.22 × 10^13^ gc/ml), the latter value being used for dilution calculations. Primers for the qPCR were: Forward 5’-AATTACCAGAGCACCCTGCC and Reverse 5’-TTTTGATCTCCCCCAGCACC.

#### 2.2.3 Intramuscular injections

**Fig. 1:**
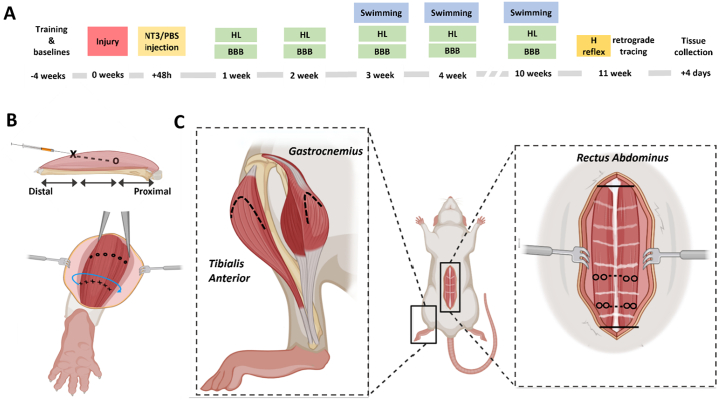
**Experimental overview.** A) Timeline indicating the timepoints of each behavioural and electrophysiological assay. BBB = Basso, Beattie and Bresnahan locomotor scale; HL = horizontal ladder. B) Top panel: cross section through a muscle indicating approximate location of needle insertion (X) and injection site and depth (O). Hindlimb muscles were injected dorsoproximally as shown. Bottom panel: circumferential arc around the muscle belly (blue), with points of needle insertion shown, designed to target the entirety of the muscle belly. C) Schematic of the rat in a supine position showing the injected muscles. Left: lateral hindlimb showing the *tibialis anterior* and *gastrocnemius*. Right: *rectus abdominus*, showing the tendinous insertions (pale pink), xyphoid process and the pubic symphysis (long solid black lines), and approximate positions of the injections (O and black dashed line). (For interpretation of the references to colour in this figure legend, the reader is referred to the web version of this article.)

Forty-eight hours after contusion, the *tibialis anterior* and *gastrocnemius* muscles of both hindlimbs as well as *rectus abdominus* were injected with AAV-NT3 or PBS (Fig. 1A). Animals were prepared similarly to above with both hindlimbs and the abdomen shaved. Injections were made using a 26G non-coring beveled needle attached to a Hamilton syringe. A total of 2.57 × 10^12^ gc of AAV-NT3 diluted in 220 μl of PBS (or equivalent amount of PBS vehicle) was injected into each animal. Targeting motor endplates was achieved by deep injections positioned in a circumferential arc across the *tibialis anterior* and *gastrocnemius* muscles (Yin et al., 2019) (Fig. 1B). For the *rectus abdominus* all injections were superficial, with care taken not to penetrate through the muscle into the peritoneal cavity.

*Tibialis Anterior:* Injections were made across the proximal aspect of the muscle, approximately two thirds the distance from the ankle to the knee. A total of 6x5μl of AAV-NT3 (3.50 × 10^11^ gc) or PBS was delivered per muscle (Fig. 1C).

*Gastrocnemius:* A total of 6x5μl of AAV-NT3 (3.5 × 10^11^ gc) or PBS were injected into both the medial and lateral heads, totalling 7.1 × 10^11^ gc in each *gastrocnemius*.

*Rectus Abdominus: Rectus Abdominus* was exposed from the xiphoid process down to the pubic symphysis. Two 5 μl injections of AAV-NT3 (4.67 × 10^11^ gc) or PBS were made between the 4th–5th and 5th–6th tendinous insertions, to target the region innervated at or below the level of thoracic contusion (Hijikata et al., 1992) (Fig. 1C). In total, 20 μl was delivered to each side.

### 2.3 Behavioural functional assessment

#### 2.3.1 Open field locomotor assessments

Locomotor functional recovery was monitored using the 0–21 point Basso, Beattie and Bresnahan scale (BBB) (Basso et al., 1995). One week prior to injury, animals were habituated to a circular Perspex open field assessment area (100 cm diameter, 20 cm high) for ten minutes each day. Testing was done in the afternoon at day −1, and then weekly following injury during a five-minute period by two assessors.

*Analysis:* The BBB score for each hindlimb were averaged between the two assessors and the BBB sub-scores similarly calculated (Popovich et al., 2012).

#### 2.3.2 Horizontal ladder

Accuracy of stepping was assessed through the animals' ability to cross a 1 m horizontal ladder with rungs spaced randomly 1-3 cm apart. Prior to injury, animals were trained to cross the ladder for 15 min periods each day and baseline values obtained within 5 days prior to injury. Testing comprised video recording three complete runs weekly following injury.

*Analysis:* Footage was slowed 5× original speed in VLC media player (VideoLan) and steps by both hindlimbs was assessed using the horizontal ladder scoring system (Metz and Whishaw, 2002). This scale assessed the accuracy of foot placement on ladder rungs categorized as fall, slip (deep or slight), corrective step (replacement, correction, imperfect partial placement), and hit (perfect placement) with each type of positioning demonstrating an ability to perform independent skilled functions. Briefly; a ‘miss’ occurs when the hindlimb fails to connect with a rung, and a ‘slip’ when the limb bears weight but slips off the rung with or without interruption to gait cycle. A “corrective step” is defined as when the hindpaw contacts the rung but is then repositioned. Finally, a ‘hit’ is a placement which results in a successful step.

#### 2.3.3 Swimming

To assess non-weight bearing locomotor function, animals swam in a rectangular glass chamber (120 cm × 12 cm × 50 cm) filled with 25 cm water at 20–23 °C (Ryu et al., 2017; Gonzenbach et al., 2010). A Perspex mirror was placed at a 45° angle on the base, allowing recording of ventral and lateral views of the animal (60 frame per second at 1920 × 1080 pixels, HERO7 Black, GoPro). Following a two-week habituation period, baseline recordings were collected within 5 days prior to injury. Rats placed at one end of the chamber would swim 80 cm, equalling one run, to an accessible platform (Fig. 3A) at the other end. Animals were assessed weekly from three weeks following injury with five complete runs, recorded during the same time of day.

*Analysis:* Hind-limb swim strokes from all runs were analysed in slow motion using VLC media player (5× original speed, VideoLan). The percentage of left-right alternating and total number of strokes was calculated for each limb. Hindlimb strokes were excluded if they: occurred outside the 80 cm assessment area; were associated with a change in direction; or were the initial stroke following an interruption to swimming. The number of coordinated hind limb strokes over all the runs performed by each animal was displayed as a proportion of total number of hindlimb strokes performed.

#### 2.3.4 H reflex recordings

Animals were anaesthetised with ketamine and medetomidine (30 mg/kg and 0.10 mg/kg i.p respectively) and treated with atropine sulphate (0.1 mg/kg s.c) and carprofen s.c (Carprieve 5 mg/kg s.c.). Body temperature was maintained at 37±1̊C using a homeothermic heat pad (Harvard Apparatus) and the ankle and hindpaw cleaned with 4% chlorhexidine. Recordings were taken from the left hindlimb at final timepoint, or from the limb with the higher BBB subscore (*n* = 4). Two 26-gauge needles were positioned, subcutaneously in parallel and approximately 2 mm apart, over the medial malleolus of the ankle to stimulate the medial plantar nerve. To record compound muscle action potentials (CMAPs) from the intrinsic hindpaw muscles innervated by the medial plantar nerve, two non-insulated needle recording electrodes were positioned at the base of first digit between the two prominent paw pads and placed superficially, 1 mm apart and 2 mm deep (Fig. 4B).

A monophasic 100 μs square wave stimulus was applied using an isolated constant current pulse stimulator (DS3, Digitimer) and the CMAP signal (amplified 2000×, band-pass filtered between 300 Hz – 6 kHz) digitized via a Power1401-3A unit (Cambridge Electrical Design Ltd., CED) and visualised in Signal software (v5, CED), recording in 5000 ms sweeps. Motor threshold was defined as the stimulus at which the M wave was elicited in three out of four stimuli. A recruitment curve was generated by recording 15 stimuli at 0.2 Hz, starting at 1× motor threshold (MT) to a maximum of 2× MT in 10% increments up to 1.5xMT and finally at 2xMT. Subsequent rate dependent depression (RDD) was assessed with a paired stimulation protocol (4) at 1.3xMT stimulus intensity. Fifteen paired pulses were delivered 300 ms into the sweep at various interstimulus intervals (ISI. Fig. 4A) Once recordings were finished, 2 mg/kg (i.p.) atipamezole hydrochloride and 5 ml saline (s.c.) was injected.

*Analysis:* Amplitude of the M and H waves was calculated in Signal software (v5, CED). The recruitment curve yielded the maximum H wave amplitude (Hmax) and maximum M wave amplitude (Mmax). For each ISI, the 15× M wave and H wave amplitudes corresponding to the conditional stimulus and the 15 for the test stimulus were pooled and averaged. These averages were then normalised to the Mmax for that testing session. Finally, RDD was calculated by representing the normalised H wave of the test stimulus as a percentage of the H wave of the conditioning stimulus.

#### 2.3.5 Retrograde tracing

Cholera Toxin Subunit B (CTb, #103, List Biologicals) was used to retrogradely trace motor neurons innervating the lateral head of the *gastrocnemius* in the left hindlimb. Animals were prepared and the *gastrocnemius visualised* as per the previous muscle injections. A total of 5x1μl of 0.5% Cholera Toxin B (diluted in ddH_2_0) was injected into the lateral muscle head, following a similar trajectory to treatment injections, using a 26G needle on a Hamilton syringe. Following surgery, animals were given 5 ml of saline and the following day a single dose of Carprieve.

#### 2.3.6 Tissue collection

Animals were deeply anaesthetized with sodium pentobarbital (Euthatal, 80 mg/kg i.p.). 1.5 ml of blood was taken through cardiac puncture of the left ventricle and stored overnight at 4 °C before processing. Animals were transcardially perfused with PBS. The left *gastrocnemius*, including both the medial and lateral heads, was removed, snap frozen, and stored at −80 °C. Animals were perfused with 4% paraformaldehyde in 0.1 M phosphate buffer. The spinal cord from C3 to the cauda equina was removed and post fixed in fresh 4% PFA in PBS for 24 h at 4 °C.

#### 2.3.7 ELISA & BCA quantification

Levels of NT3 protein in the serum were assessed using a Human Neurotrophin-3 ELISA Kit (#ab100615 Abcam), which can detect vector-derived and endogenous NT3. Whole blood was centrifuged (7200 *g*, 25̊C), the serum removed and stored at −80 °*C. ELISA* was performed as per the manufacturer's instructions and the plate read immediately at 450 nm using a BMG LabTech FLUOStar (Omega). NT3 levels were normalised to the total amount of protein extracted from each sample determined by a Bicinchoninic acid assay (BCA) assay (#71285–3, Novagen, Millipore). Serum samples were diluted 1:150 in PBS, and a four-parameter standard curve was generated (above *r* > 0.99).

#### 2.3.8 Ex vivo *MRI*

For lesion quantification the epicentre of the lesion was imaged using a 9.4 T MRI scanner (Brucker Biospec). T2 weighted (T2W) images were acquired using a fast spin-echo sequence: echo train length = 4, effective TE = 38 ms, TR = 3000 ms, FOV = 40 × 20 × 20 mm, acquisition matrix = 400 × 200 × 200, acquisition time = 9 h 20 m. A custom-made device enabled simultaneous scanning of the T6-L1 regions of 14 spinal cords. Tissue was fully submerged in Fomblin (Solvay) and all air bubbles removed prior to scanning. The spinal cords were prepared for scanning as described previously (Sydney-Smith et al., 2021a) with additional care taken due to the reduced stability of the spinal cord around the epicentre.

*Analysis:* Convert3D (ITK-SNAP) was used to alter the voxel dimensions to isotropic voxel size of 50 μm. The composite image was separated into individual spinal cords to final voxel dimensions of 30x100x30μm in the *X* x *Y* x *Z* axis respectively. Within ITK-SNAP, the contrast was increased (minimum adjustment:0.03, maximum adjustment:0.82, levels:0.41, window:0.82) and automatic segmentation performed for: presumptive spared white matter (lower limit = 0.18, upper limit = 0.28) and total spinal cord (lower limit = 0.00, upper limit = 0.29). The segmentation parameters were: radius 2.5, competition force =1.000, smoothing force = 0.2, α = 1.000, β = 0.100, speed = 1.00. All parameters were identical for each spinal cord. A screenshot series of the transverse views of the mask and images every 100 μm were quantified in FIJI 9 (U.S. National Institutes of Health). Volume for each parameter was calculated as the thickness of the voxel multiplied by the cross-sectional area. The cross-sectional area of the tissue was measured in a region 4.1 mm in length and encompassing the epicentre.

#### 2.3.9 Immunohistochemistry for VGlut1

Following ex vivo MRI, spinal cords were washed in PBS, cryoprotected in 30% sucrose, frozen in OCT compound and cut in transverse 15 μm sections. Sections were immunostained using; rabbit anti-vGlut1 antibody (1:1000, #135302, Synaptic Systems), Goat-anti-CTB (1:2000, #703, List Biolgicals), Mouse-anti-NeuN (1:500, #MAB377, Millipore). Sections were counterstained with Alexa 546 and Alexa 488 (1:1000, #A10040 and #A10036, Thermofisher) and Dylight 650 (1:500, #ab96938, Abcam). Five sections throughout the L4-L6 segments were selected and traced α-motor neurons (Ctb+/ NeuN+) in the ventral horns were imaged at 400× magnification (LSM 710, Zeiss). Animals with fewer than five traced motor neurons were excluded from analysis.

*Analysis:* Analysis was performed in FIJI (NIH) by measuring raw integrated density within a 2.5 μm band surrounding the soma, and manually counting distinct vGlut1+ puncta abutting the motor neuron.

### 2.4 Statistical analysis

Prior to all experiments, power analysis (G*Power (version 3.1), type 1 error threshold (α) ≤ 0.05 and power (1-β) ≥ 0.90) was conducted to ensure reliable data based on previous experiments. Statistical analysis was performed using either Prism (version 9, GraphPad) or SPSS (version 25, IBM). Locomotion behavioural assessments were analysed using a linear model with a suitable covariance structure as described previously (Duricki et al., 2016c). For analysis of the horizontal ladder, for each dependent variable (e.g., Slips, Hits or Miss) in turn, we specified a linear mixed model in which we included limb (Left or Right) as a factor. Because these analyses often revealed a difference between limbs (Supplementary Table 1), we also analysed each limb separately and report these values in the Results section.

All data sets were found to be normally distributed by viewing frequency histograms and/or satisfying Kolmogorov Smirnov and Shapiro-Wilk tests. Error bars in graphs are mean ± Standard error of mean. Divergences were considered significant if *P* < 0.05. In all figure panels: NT3 = blue triangles, controls = orange squares, sham = black diamonds, * = P < 0.05, ** = *P* < 0.01, *** = *P* < 0.001, and **** = *P* < 0.0001. If no post-hoc result is shown, comparison was not significant.

## 3 Results

### 3.1 NT3 treatment recovered skilled motor function and coordination following injury

**Fig. 2:**
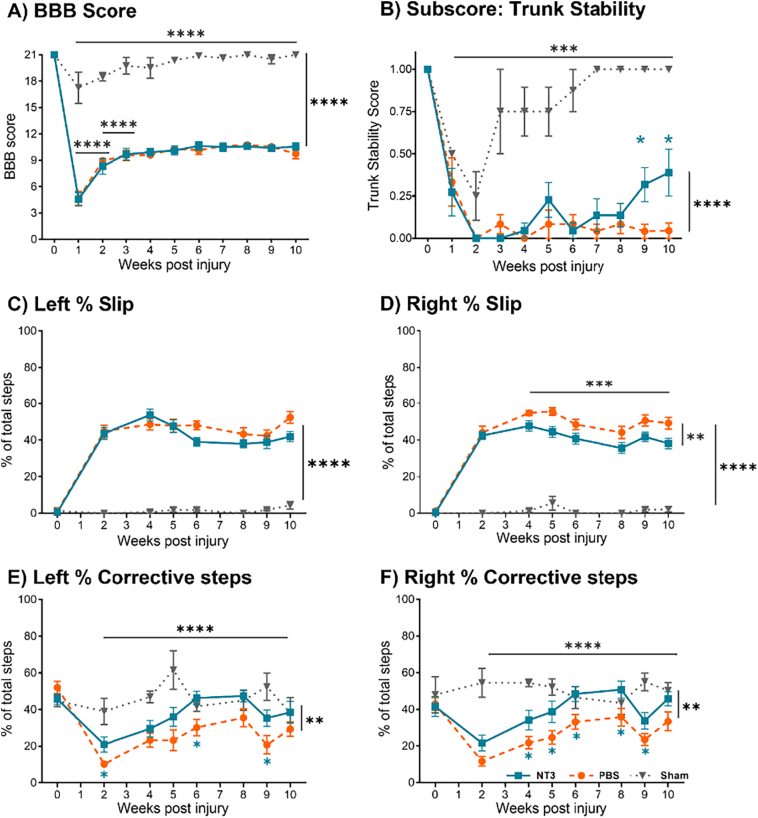
**NT3 treatment can improve specific, skilled hindlimb functions.** A) T9 contusion reduced the BBB score of all animals. NT3 treatment failed to cause recovery in total locomotive function compared to controls. Sham injured animals showed no sustained deficit in hindlimb function. B) Analysis of BBB sub-scores showed that trunk stability was impaired after injury compared to sham. NT3 treatment increased trunk stability at 9- and 10-weeks post injury compared to PBS controls. C-D) NT3 treated animals showed a similar proportion of slips to PBS treated animals on the horizontal ladder with the C) left hindlimb and a decrease from 4 weeks post injury with the D) right hindlimb. *E*-F) Similarly both hindlimbs of NT3 treated animals showed an increase in skilled corrective stepping following misplacement compared to the PBS group. Their performance was comparable to sham animals by week 10. Baseline recordings were not used as a covariate but were excluded for tests of effects and interactions. For all panels: NT3 (blue squares) *n* = 11, for PBS (orange circles) n = 11, for sham (grey triangles) *n* = 4. (For interpretation of the references to colour in this figure legend, the reader is referred to the web version of this article.)

A contusion injury of 250kdyn at the T9 spinal level produced a severe SCI in 23 adult female Wistar rats. Animals were randomised into either an NT3 group (*n* = 11, receiving a total of 2.57 × 10^12^ gc of AAV-NT3 in 220 μl PBS) or a control group (*n* = 12, receiving 220 μl PBS). Uninjured rats served as the sham control (sham group, *n* = 4) and received all surgical procedures and after care apart from the displacement and the muscle injections. No differences in the force (NT3 = 256 ± 4.7 kDyn, PBS =255 ± 3.3 kDyn) or displacement (NT3 = 1417 ± 157 μm, PBS = 1428 ± 147 μm) of the spinal cord were observed between the control and treatment groups (Unpaired *t-*test, Force *p* = 0.63, *t* = 0.49; displacement *p* = 0.86, *t* = 0.18). Behavioural assessments (BBB, horizontal ladder, and swimming) determined whether NT3 treatment improved locomotor function (Fig. 1). While hindlimb locomotion across the open field was impacted one week following trauma (BBB; Fig. 2A; Supplementary Fig. 1A; effect of group, linear model, F(2,24) = 106, *p* < 0.0001, post hoc LSD comparison, NT3 vs Sham p < 0.0001, PBS vs Sham p < 0.0001), AAV-NT3 did not improve gross locomotive function compared to injured controls (Fig. 2A; post hoc NT3 vs PBS *p* = 0.92, effect of time x group, linear model, F(18,213) = 1.2, *p* = 0.24). Both groups plateaued in recovery at three weeks post injury (subsequent weeks vs 10 weeks post injury *p*-values>0.09). Sham animals showed a transient effect on locomotor function due to the laminectomy surgery. Assessment of BBB subscores showed injured animals regained some locomotive capacity following injury (Supplementary Fig. 1B; effect of time post injury, linear model, F (2, 25) = 184.0, *p* < 0.0001, post hoc LSD comparison, 1 week vs 4 weeks *p* = 0.0007, 4 weeks vs 9 weeks *p* = 0.01, 9 weeks vs 10 weeks *p* = 0.02) and a consistent but non-significant trend for the NT3 group to exhibit higher average functional capacity compared to controls (effect of group, linear model, F(2,25) = 180, *p* < 0.0001, post hoc LSD comparison, NT3 vs Sham p < 0.0001, PBS vs Sham p < 0.0001, NT3 vs PBS *p* = 0.08). This trend appeared in both the left and right hindlimb of NT3 treated animals (Supplementary Fig. 1C; effect of group, linear model, F(5,50) = 139.1, *p* < 0.0001, post hoc LSD comparison, NT3 right hindlimb vs. PBS right hindlimb *p* = 0.21, NT3 left hindlimb vs. PBS left hindlimb *p* = 0.14). Subscore analysis did reach significance for paw position at 4 weeks, but this was not maintained (Supplementary Fig. 1E; effect between group x time post injury, linear model, F(2,230) = 5.25, p < 0.0001, post hoc LSD 4 weeks post injury NT3 vs Sham p < 0.0001, PBS vs Sham p < 0.0001, NT3 vs PBS *p* = 0.014). Toe clearance improved following injury (effect of time, linear model, F (2, 25) = 125, p < 0.0001, post hoc LSD comparison, 1 week vs 3 weeks *p* = 0.027, 3 weeks vs 9 weeks *p* = 0.03) but no effect of NT3 treatment was detected (effect of group x time post injury, linear model, F(2,235) = 15, p < 0.0001, post hoc LSD comparison, NT3 vs Sham p < 0.0001, PBS vs Sham p < 0.0001, NT3 vs PBS *p* = 0.18). NT3 treatment improved trunk stability at later time points of nine- and ten-weeks post injury (Fig. 2B; post-hoc LSD NT3 vs PBS *p* = 0.090, effect between group x time post injury, linear model, F (18,213) = 2.78, *p* = 0.0002, post-hoc LSD NT3 vs PBS at 9 wpi *p* = 0.024, at 10 wpi *p* = 0.041).

To assess restoration of skilled hindlimb function animals were assessed stepping on a horizontal ladder from two weeks post injury (Fig. 2C-F). Contusion caused a sustained drop in the proportion of error-free steps by both hindlimbs (Supplementary Table 1; Supplementary Fig. 2A-B; effect of group, linear model, Left = F(2,23) = 910, *P* < 0.0001, Right = F(2,24) = 693.6, P < 0.0001) and increased the number of missed steps (Supplementary Fig. 2C-D; effect of group, linear model, Left F(2,24) = 9.95, *P* = 0.0007, Right = effect of group, linear model, F(2,24) = 5.68, *P* = 0.0095). NT3 treatment reduced the number of slips overall (considering both hind limbs together; Supplementary Table 1) and this reached significance on the right but not on the left (Fig. 2C-D; effect of group, linear model, left = F(2,24) = 120.6, P < 0.0001, post hoc NT3 vs PBS *p* = 0.12, right = F(2,24) = 111.5, P < 0.0001, NT3 vs PBS *p* = 0.0027). NT3 treated rats corrected more mis-steps than PBS treated rats, recovering to the level of Sham injured animals from six weeks post injury (Fig. 2E-F; effect of group, Linear model, Left = F(2,24) = 6.75, *P* = 0.0047, post hoc LSD for NT3 vs Sham *p* = 0.13, PBS vs Sham *P* = 0.0024 and NT3 vs PBS *p* = 0.019, Right = F(2,24) = 7.6, *P* = 0.0026, post hoc LSD for NT3 vs Sham *p* = 0.089, PBS vs Sham *P* = 0.0012 and NT3 vs PBS *p* = 0.016). These data suggested that NT3 treatment could enhance specific aspects of hindlimb function to aid skilled locomotion.

### 3.2 NT3 treatment restores coordinated limb movement during swimming

**Fig. 3:**
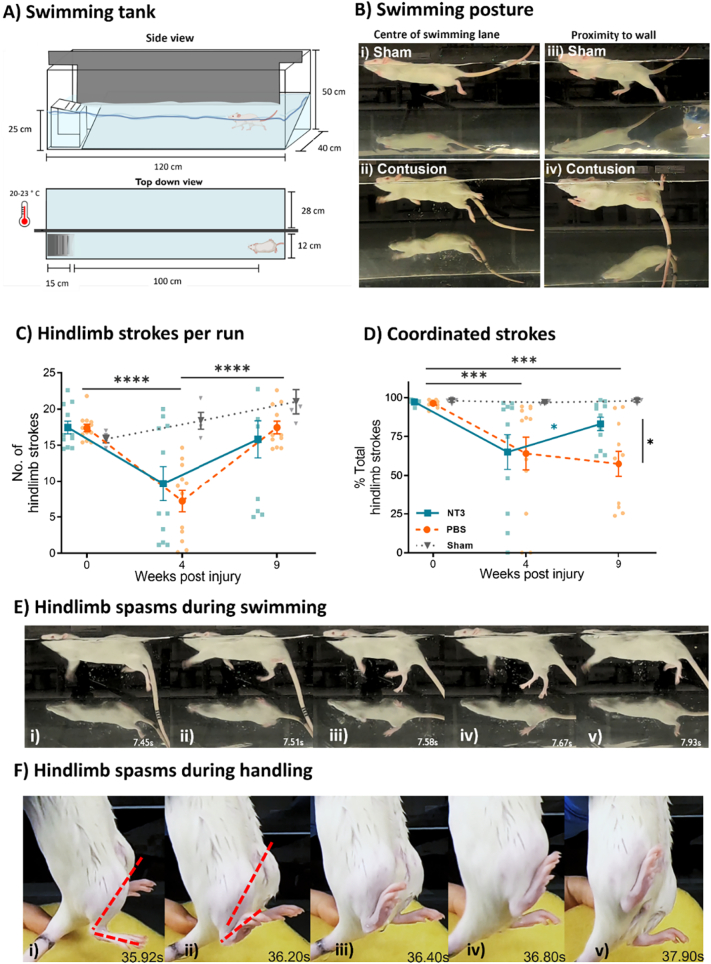
**NT3 treatment reverses deficits in hindlimb coordination during swimming**. A) Swimming tank schematic. A 45° mirror provided simultaneous lateral and ventral views. Water temperature = 20–23 °C. B) Representative images showing swimming posture between i + iii) sham and ii + iv) contused animals (3 wpi) either i + ii) in the swimming lane or iii + iv) close to the tank walls. Note the pelvic girdle rotated around the rostral caudal axis. C) The sum of left and right hind limb strokes. D) The proportion of alternating left:right hindlimb strokes following treatment. **E)** Representative images showing a 0.5 s bilateral hindlimb spasm during open swimming **where i)** cyclic hindlimb strokes were ii) abruptly stopped, iii-iv) limbs extended, and then v) normal function resumed. F) Representative images showing the progression of a 3 s unilateral dorsiflexion spasm of the right hindpaw following swimming. Dotted red line i-ii) shows the closure of the angle made between the dorsal surface of the hindpaw and the shin. Adduction of the hip joint was also present, shown by iv) the inward rotation of the right hindlimb. (For interpretation of the references to colour in this figure legend, the reader is referred to the web version of this article.)

To see if NT3 treatment facilitated motor tasks that do not require weight support, animals were assessed during an 80 cm swim in a customised tank kept between 20 and 23 °C to avoid temperature-evoked spasms (Fig. 3A) (Gonzenbach et al., 2010). At baseline, animals consistently used the hindlimbs for propulsion in swimming with forepaws kept close to the jaw (Figure 3Bi). Contused animals were able to swim but showed abnormal use of the forelimbs for propulsion with the tail submerged (Figure 3Bii). These abnormalities are consistent with previous studies (Smith et al., 2006; Xu et al., 2015) (Fig. 3 Biii-iv).

The effects of injury on swimming gait are reflected in the quantification of animals' swimming activity. The number of hindlimb strokes four weeks after injury was reduced by approximately 50% (Fig. 3C; baseline: 16.6 ± 2.2, NT3: 9.6 ± 7.8, PBS: 7.2 ± 5.1, and Sham: 18.3 ± 2.3 strokes) which recovered by nine weeks post injury (NT3: 15.9 ± 8.8, PBS:17.4 ± 2.8, Sham: 21.0 ± 3.3 strokes; effect of time, linear model including all groups, F(1.52,35.8) = 13.32, *p* = 0.0002, post hoc LSD, baseline vs 4wpi *p* < 0.0001, 4wpi vs 9wpi p < 0.0001, baseline vs 9wpi *p* = 0.92). However, at no stage was there a difference between treatment groups (effect of group, linear effects model, F(2,24) = 2.13, *p* = 0.15). Contusion injury caused a marked reduction in hindlimb coordination, with a lower proportion of strokes following left-right rhythmicity (Fig. 3D; effect of time, linear model, F(1.52,35.75) = 7.15, *p* = 0.005, post hoc LSD, baseline vs 4wpi *p* = 0.0003, baseline vs 9wpi p < 0.0001, 4wpi vs 9wpi *p* = 0.36). Over time, NT3 treatment caused an increase in the proportion of coordinated strokes similar to sham animals at nine weeks, whilst PBS treated animals had no improvement (effect of group x time, linear model F(4,47) = 2.57, *p* = 0.48, post hoc LSD at week 9, NT3 vs PBS *p* = 0.013; NT3 v Sham *p* = 0.272; and PBS v Sham *p* = 0.0041). These data similarly suggest that NT3 treatment could enhance specific aspects of hindlimb function aiding coordinated movement.

### 3.3 Contusion did not cause spasms or hyperreflexia of the propriospinal circuity

Only one animal showed noticeable spasm, clonus or otherwise abnormal activation of the hindlimbs or trunk during swimming (during one single instance; Fig. 3E) and only five animals showed presumptive spasm activity once following swimming (Fig. 3F). Further, the animals did not show any spontaneous spasm during meticulous observation during behavioural tests, when in the open field, and in their home cage. The rareness of spasms was unexpected as this is activity associated with this injury (Ryu et al., 2021).

**Fig. 4:**
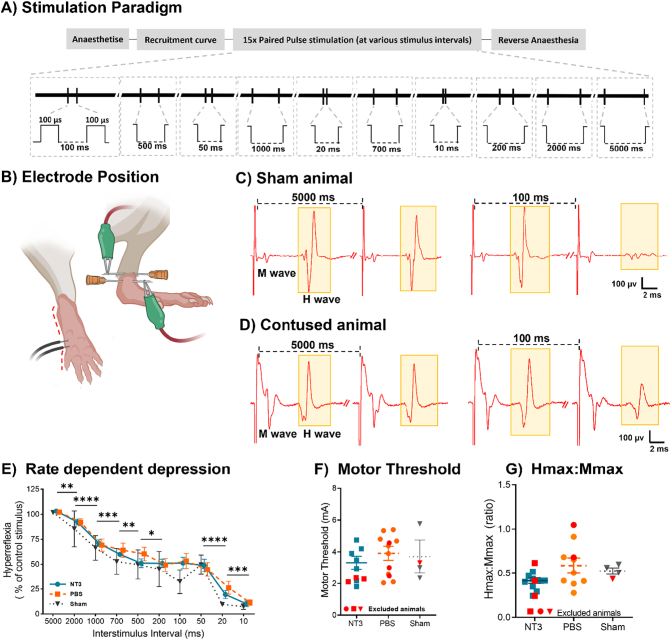
**250kdyn thoracic contusion did not generate hyperreflexia.** A) Stimulation protocol. B) Position of the recording and stimulation electrodes; note that recording and stimulation electrodes were placed in the same limb; two images show the same limb from different angles. C&D) Representative traces from and contused animals highlighting the M and H wave (yellow) associated with the test stimulus at either 5000 ms or 100 ms ISI. E) Rate dependent depression was the same regardless of animal group. F) Motor thresholds were similar between groups (red symbols denote excluded animals). G) The Hmax:Mmax ratio was comparable between groups. (For interpretation of the references to colour in this figure legend, the reader is referred to the web version of this article.)

To investigate hyperreflexia, the H reflex of the animals was recorded from the hindpaw at 11 weeks post injury during paired stimulation of the medial plantar nerve over a defined range of frequencies (Fig. 4A-B) (Smith et al., 2018; Lee-Kubli and Calcutt, 2014; Chang et al., 2019; Kathe et al., 2016). The amplitude of the normalised H wave from the conditional stimulus remained comparable to the H wave from the test stimulus in all three groups (NT3 = 103.1 ± 5%, PBS 102.2 ± 2.6%, sham 101.6 ± 1.3%; Fig. 4C-E). Animals were excluded from analysis if they received a higher dose of anaesthetic prior to recording or showed limited rate dependent depression (RDD) from 2000 ms to 50 ms (as occurred in one sham animal). This did not affect the conclusions drawn (Supplementary Fig. 3A-H), as levels of rate dependent depression remained similar (Supplementary Fig. 3A, effect of group, linear model, F(2,22) = 0.14, *P* = 0.87).

RDD was comparable between injured and sham groups (Fig. 4E; effect of group, linear model, F(2,15) = 0.83, *p* = 0.45). RDD increased with shorter interstimulus interval (ISI, effect of interstimulus interval, linear model, F(1.9,27.6) = 64.1, *P* < 0.0001, 5000 ms vs 2000 ms *p* < 0.0001, 2000 ms vs 1000 ms *p* ≤0.0001, 1000 ms vs 700 ms *p* = 0.0007, 700 ms vs 500 ms *p* = 0.095, 500 ms vs 200 ms *p* = 0.021, 200 ms vs 100 ms *p* = 0.73, 100 ms vs 50 ms *p* = 0.81, 50 ms vs 20 ms p < 0.0001, 20 ms vs 10 ms *p* = 0.0046). Sham animals had a consistent trend towards smaller H wave amplitudes at each ISI. The motor threshold was unaltered following injury and treatment (Fig. 4F; one-way ANOVA, F(2,17) = 0.42, *p* = 0.65). Multiple electrophysiological readouts remained comparable; maximum M wave amplitude (Supplementary fig. 3D, one-way ANOVA, F(2,21) = 1.86, *p* = 0.18), maximum H wave amplitude (Supplementary fig. 3E, one-way ANOVA, F(2,21) = 0.50, p = 0. 61). The ratio between the Hmax and Mmax values, which can indicate excitability of the reflex, was unaltered after injury (Fig. 4G, one-way ANOVA, F(2,21) = 2.41, *p* = 0.11). No differences occurred following exclusions of some rats for Hmax:Mmax (one-way ANOVA (F(2,15) = 1.435, *p* = 0.23), Mmax (Supplementary fig. 3G, one-way ANOVA, F(2,15) = 0.9, *p* = 0.41) and Hmax (Supplementary fig. 3H, one-way ANOVA, F (2,15) = 0.07, *p* = 0.93). Overall, these results indicate that a lumbar H reflex was not detectably altered by severe thoracic contusion.

### 3.4 Intramuscular injection of AAV1-CMV-NT3 elevated NT3 serum levels

**Fig. 5:**
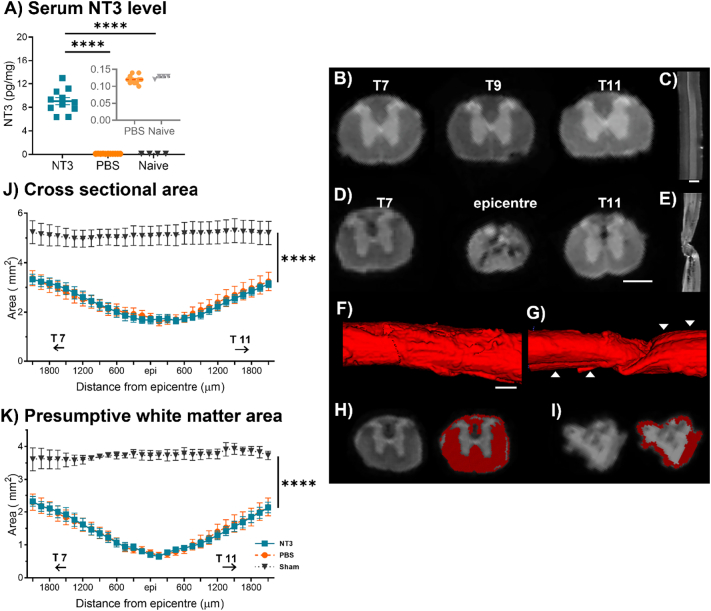
**Intramuscular injection of AAV-NT3 increased circulating NT3 levels but did not alter lesion volumetrics.** A) Intramuscular injection of AAV-NT3 resulted in an elevation of NT3 protein in serum in all treated animals. SCI followed by intramuscular injections of PBS did not alter the level of NT3 in serum, shown in insert (post hoc LSD, PBS vs Sham *p* = 0.99). B) Transverse sections through an intact spinal cord at T7, T9, and T11, showing the characteristic grey and white matter morphology. C) Sagittal view of the intact cord. D) Transverse sections of a chronically contused spinal cord at T7, T9, and T11 demonstrating hypointense imaging in the lesion epicentre. E) Sagittal section of the injured cord where the lesion epicentre is apparent from the reduced width of the spinal cord and loss of grey/ white matter distinction. Hypointense signal was seen extending caudally from the epicentre. F&G) Sagittal, volumetric renderings of the F) intact and G) injured spinal cords (T7 through T11). Occasional artefactual twisting of the epicentre was visualised by tracing the ventral fissure, indicated by arrow, as it courses caudally through the lesion. H&I) T2 Weighted MR images showing H) T7 and I) the epicentre with the corresponding automatically generated mask of presumptive spared white matter. J) Total cross-sectional area of the spinal cord, inclusive of spared tissue and cavity, throughout the lower thoracic spinal cord. K) Presumptive spared white matter through the thoracic spinal cord following treatment.

ELISA was performed on serum collected at 11 weeks post injury to quantify levels of circulatory NT3 protein, expressed as a proportion of total protein content in each sample. Injection of AAV-NT3 into gastrocnemius, tibialis anterior and rectus abdominus muscles elevated circulating NT3 levels (Fig. 5A; NT3 = 9.05 ± 0.61 pg/mg, PBS = 0.12 ± 0.004 pg/mg, Sham = 0.13 ± 0.003 pg/mg; effect of group, one-way ANOVA, F(2,23) = 143.2, *p* < 0.0001, post hoc LSD NT3 vs PBS p < 0.0001, NT3 vs Sham p < 0.0001), demonstrating that the AAV was producing NT3. There was no correlation between NT3 serum levels 11 weeks post injury with the animal's functional recovery shown through the horizonal ladder at 10 weeks post injury (Pearson's Rank Correlation; <0.05 for “miss”, “slip”, “hit” or “corrective step”; data not shown).

### 3.5 NT3 treatment did not affect lesion volume

Ex vivo Magnetic Resonance Imaging (MRI) was used to acquire high resolution T2 weighted (T2W) images of the thoracic spinal cord (Fig. 5B-I) following termination of the experiment to assess any differences in lesion volume between groups. At this resolution, there was a clear distinction between the grey and white matter from T7 to T11 on both sham (Fig. 5B-C&F) and contused cords (Fig. 5D-E&G). The epicentre was determined as the spinal cord transverse section with the smallest cross-sectional area of presumptive spared white matter. Within the epicentre, there was a mixture of fragmented hypointense signal, likely corresponding to fluid filled cavitation, interspersed with scar tissue and/or oedema. Sagittal sections showed that some injured spinal cords were deformed at the contused region (Fig. 5E&G), likely twisted during preparation for scanning, but time constraints due to COVID-19 lockdown precluded reimaging of those cords.

Total spinal cord transverse cross-sectional area and presumptive spared white matter were measured following thresholding for the respective tissue and automatic detection in ITK-SNAP software. Presumptive spared white matter was defined as regions with a T2 weighted signal comparable to, and continuous with, the signal in the lateral funiculus in the rostral and caudal most spinal level imaged (Fig. 5H-I). Injury reduced the total transverse cross-sectional area in the regions extending at least 2 mm rostral and caudal from the lesion epicentre (Fig. 5J; effect of group, two-way RM ANOVA, F(2,23) = 29.4, *p* < 0.0001; NT3 vs Sham p < 0.0001, PBS vs Sham p < 0.0001). The total transverse cross-sectional area at the epicentre was reduced by 68% in the NT3 group compared to the equivalent spinal cord level in Sham group animals, and by a similar amount in the PBS group (NT3 = 1.68 ± 0.5 mm^2^, PBS = 1.61 ± 0.5 mm^2^ and Sham = 5.10 ± 0.5 mm^2^; effect of group x level, two-way RM ANOVA, F(56, 637) = 2.65, p < 0.0001, rostral-most region; NT3 vs Sham p < 0.0001, PBS vs Sham p < 0.0001, at epicentre; NT3 vs Sham p < 0.0001, PBS vs Sham p < 0.0001, NT3 vs PBS *p* = 0.86 and caudal-most region NT3 vs Sham p < 0.0001, PBS vs Sham p < 0.0001). Contusion reduced presumptive spared matter at least 2 mm rostral and caudal to the epicentre (Fig. 5K, effect of group x level, two-way RM ANOVA, F(56,612) = 2.49, p < 0.0001, post hoc LSD rostral-most region NT3 vs Sham p < 0.0001, PBS vs Sham p < 0.0001; at epicentre, NT3 vs Sham p < 0.0001, PBS vs Sham p < 0.0001; and caudal-most region NT3 vs Sham *p* = 0.0002, PBS vs Sham p < 0.0001). At the lesion epicentres of both PBS and NT3 treated groups, presumptive spared white matter was reduced by 36% compared to Sham (NT3 = 0.64 ± 0.17 mm^2^, PBS = 0.67 ± 0.30 mm^2^ and Sham = 3.7 ± 0.2 mm^2^; post hoc LSD NT3 vs PBS *p* = 0.94). These data suggest that NT3 treatment did not reduce lesion volumes.

### 3.6 NT3 normalised vGlut1+ excitatory input onto motor neurons

**Fig. 6:**
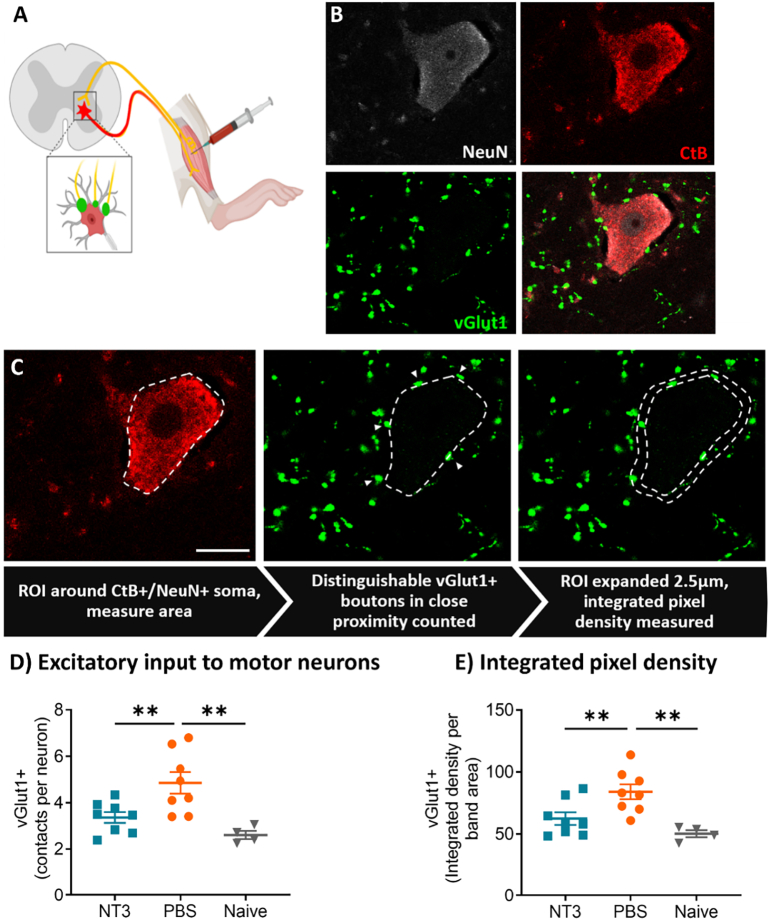
**Afferent derived excitatory input was normalised by NT3 treatment**. A) Motor neurons innervating the lateral head of the left *gastrocnemius* were retrogradely traced with intramuscular CTb injection to target the motor end plates. Ia afferent fibres innervating the lateral *gastrocnemius* make direct vGlut1+ contacts with these homonymous motor neurons. B) Numerous vGlut1 puncta were present on both the soma compartment and proximal dendrites of traced α-motor neurons (CTb+/ NeuN+). C) Overview of the methods used to quantify vGlut1+ puncta on motor neuron. Arrow heads mark distinct vGlut1 puncta which were abutting the soma membrane and counted. D) T9 contusion increased individual vGlut1+ puncta abutting CTb+/NeuN+ soma, which was normalised back to levels comparable with uninjured animals by NT3 treatment. E) NT3 was able to normalise the increase in integrated density of vGlut1+ signal within a 2.5 μm band around the soma after thoracic contusion. For all panels, scale bar = 20 μm. Animals per group: Sham = 4, NT3 & PBS = 8. Total number of motor neurons analysed: Sham = 58, NT3 = 111 and PBS = 117, Number of Puncta: Sham = 153, NT3 = 338 and PBS = 556.

To determine treatment induced proprioceptive sprouting, immunohistochemistry was used to assess direct glutamatergic (vGlut1+) connections from Ia afferent fibres on α-motor neurons. Following all assessments, motor neurons innervating the lateral head of the *gastrocnemius* were retrogradely traced with Cholera Toxin B (CtB, Figs. 1A& 6A). The L4-L6 spinal cord region, encompassing the *gastrocnemius* motor pool (Mohan et al., 2014), was stained for vGlut1, a marker of Ia afferent derived motor neuron input and CTb to identify motor neurons innervating the lateral head of the *gastrocnemius* (Fig. 6B). NeuN was used as an additional marker to exclude connections onto homonymous γ-motor neurons (Friese et al., 2009).

vGlut1+ immunoreactive puncta seen on the motor neuron perimeter were quantified: 1) as individual counts and 2) as integrated pixel intensity. A near doubling of vGlut1+ puncta close to motor neuron somas was observed following contusion injury (average puncta in Sham = 2.6 ± 0.17, PBS = 4.9 ± 0.45 puncta; Fig. 6D). However, NT3 treatment was able to reduce this increased marker of excitatory input back to a level comparable with Sham animals (NT3 group: 3.4 ± 0.23 puncta; one way ANOVA, F(2,17) = 8.75, *P* = 0.0024, post hoc LSD; NT3 vs PBS *p* = 0.0063, NT3 vs Sham *p* = 0.21, PBS vs Sham *p* = 0.0014). A similar result was detected considering integrated pixel intensity (Fig. 6E; one way ANOVA, F(2,17) = 8.47, *P* = 0.0028, post hoc LSD; NT3 vs PBS *p* = 0.0083, NT3 vs Sham *p* = 0.19, PBS vs Sham p = 0.0014). These results indicate that intramuscular NT3 treatment normalised the number of vGlut1+ puncta on hindlimb motor neurons after contusion injury.

## 4 Discussion

The aim of this study was to determine whether NT3, delivered with a clinically feasible delay to the muscles of the ankle, *tibialis anterior* and *gastrocnemius*, and lower portion of the *rectus abdominus* following a T9 contusion, would modulate the lumbar neural circuitry facilitating functional recovery. We demonstrated that treated animals had a 75-fold increase in circulating NT3 levels at 11 weeks post injury. Animals did not demonstrate gross improvement in behavioural function but showed, through numerous motor tasks, that the treatment strategy enhanced skilled hindlimb stepping, coordination of these limbs when swimming and trunk stability in the open field. We suggest that this increase in function is due to modulated proprioceptive input innervating these muscles. Interestingly, the injury did not result in measurable signs of spasticity, such as hindlimb spasms and hyperreflexia, suggesting that these typical SCI outcomes are not reliably induced through severe thoracic contusion. This work demonstrates that a clinically feasible 48-h delay in AAV1-NT3 treatment application can help restore fine functional motor control and supports its use as a therapeutic strategy for SCI.

We used an AAV1-CMV mediated gene therapy to deliver a therapeutic transgene via injection into peripheral muscle. This is an effective and safe delivery system used previously in human patients in the Glybera therapy and also in numerous preclinical studies (Sydney-Smith et al., 2021b; Kakanos and Moon, 2019; Wang et al., 2018; Zhou et al., 2003; Chen et al., 2006; Fortun et al., 2009; Petruska et al., 2010). NT3 is safe and well-tolerated in human adults when given peripherally at high doses (Sahenk et al., 2005; Parkman et al., 2003; Coulie et al., 2000) and an AAV-tMCK-NT3 treatment applied through injection into the leg muscle has recently been approved for use in a phase 1 clinical trial (NCT03520751). These data would suggest that the AAV1-CMV-NT3 vector we used would be safe for use in clinical patients. However, future work will introduce an off switch in the AAV vector to inhibition long-term transgene expression in case of safety concerns (Nguyen et al., 2021; Zhong et al., 2020). Here, we confirmed the AAV serotype 1 produced high quantities of circulating NT3 long after initial injection. We have previously shown that AAV1-NT3 injection into muscle elevates protein levels of NT3 specifically in the treated muscles, their ipsilateral dorsal root ganglia (DRG), and spinal cord dorsal horn and motor neurons as well as in the blood (Kathe et al., 2016; Duricki et al., 2016a). Our work, and that of others, has shown that following intramuscular or intraneural injection of AAV1-CMV-NT3, retrograde transport of NT3 protein from muscle to DRG, large sensory and motor neurons can occur (Curtis et al., 1998; Distefano et al., 1992; Kathe et al., 2016; Han et al., 2019; Wang et al., 2018).

### 4.1 Ex vivo imaging of the spinal cord showed an extensive injury was produced

Ex vivo MRI revealed an extensive lesion. Several comparative studies have shown that quantification of injury size from MRI closely matches that from conventional histology (Ditor et al., 2008; Byrnes et al., 2010; Mihai et al., 2008). We report sparing of the lateral and ventral white matter likely encompassing the reticulospinal tract (Schucht et al., 2002), propriospinal axons (Flynn et al., 2017; Sheikh et al., 2018) and possibly the rubrospinal tract (Morris and Whishaw, 2016). The corticospinal tract, providing fine motor control, is predominantly located in the dorsal columns, which have considerable white matter loss. Tracing experiments after a similar severity of contusion to the cervical spinal cord disrupts all but a few CST fibres in the dorsal lateral white matter (Anderson et al., 2009), and given the smaller diameter of the thoracic cord impacted in this study, it is expected that even fewer CST fibres remain caudal to injury in our animals.

### 4.2 NT3 treatment promoted recovery in skilled control of the hindlimb

Our results showed a beneficial effect of NT3 treatment on trunk stability, swimming coordination, and stepping accuracy. The contusion produced a severe motor deficit from which, despite some endogenous recovery, the animals were unable to regain consistent plantar stepping and interlimb coordination. This is consistent with previous studies following moderate thoracic contusion from a weight drop impactor (Bose et al., 2012; Basso et al., 1995). The locomotor impairments we report were, unexpectedly, not as severe as studies using a 250 kDyn contusion at T7 (Park et al., 2016), T8 (Ryu et al., 2017) or T9 spinal levels (Chang et al., 2019), where injured animals had limited hindlimb movement early in recovery and plateaued from week four, being unable to perform weight-supportive plantar stepping (e.g., at BBB=9 in Chang et al., 2019 versus BBB=10 in ours). These subtle differences between our study and theirs may suggest the contusion performed here did not induce as severe a neurological deficit as expected despite similar force of contusion. Similarly, the functional improvements we saw following NT3 treatment were not as extensive as others have demonstrated despite the same dose of AAV-NT3 being used for hindlimb muscles (Chang et al., 2019) and considering that we additionally treated *rectus abdominis* muscles. This may suggest that delivery of NT3 at 24 h is superior to delivery at 48 h. This should be investigated in further experiments.

Data regarding toe clearance and trunk stability was of particular significance to the study as NT3 was applied to the main ankle flexor and extensor muscles and the rectus abdominus muscles. NT3 treated animals consistently scored higher averages above PBS treated rats for toe clearance and we identified an effect of NT3 treatment on trunk stability during open field walking. Numerous muscles are involved in trunk stability including the *rectus abdominus*, *obliques* and muscles of the vertebral column such as *erector spinae* (Musienko et al., 2014). Here only the caudal half of the *rectus abdominus* was injected with AAV-NT3 (although NT3 circulating in the blood might have affected non-injected neuromuscular circuits). Improved trunk stability has been achieved after thoracic contusion experiments following stair training (Singh et al., 2011). These data may suggest that NT3 treatment can mimic some of the effects of regular training in specific circumstances. Further experimentation where all muscles involved in trunk stability are injected with NT3 should be considered.

The effect of NT3 improving precision hindlimb stepping on the horizontal ladder is consistent with other work (Duricki et al., 2016a; Kathe et al., 2016; Fortun et al., 2009). This behavioural effect occurred at two weeks following treatment administration, indicative of the rapid expression (Zincarelli et al., 2008; Dang et al., 2017; Petruska et al., 2010) and effect of the transgene. We demonstrate the ability of the NT3 to aid the animals' ability to correct misplaced steps, while other skilled functions (e.g. accurate initial placement) is not improved. This may suggest restoration of function through proprioceptive afferents which relay information regarding inappropriate positioning during weight baring steps. Administration of a viral vector encoding NT3 into the sciatic nerve partially recovered error free hindlimb stepping following thoracic contusion (Han et al., 2019), likely a result of NT3 mediated regrowth of dendritic arbors of caudal motor neurons. This was accompanied by elevated numbers of synaptic-like contacts from long descending propriospinal neuron terminals onto motor neuron soma and dendrites. It is possible the recovery we see in hindlimb performance during multiple behaviours is due to a similar enhancement of spinal connectivity since retrograde transport of NT3 occurs from peripheral tissues to motor neurons (DiStefano et al., 1992; Kathe et al., 2016). NT3 mediating connectivity to these circuits may explain the improved coordination of hindlimb strokes and postural corrections, as contralaterally projecting propriospinal interneurons or commissural V0 interneurons may be affected (Talpalar et al., 2013). As such dissection of the role NT3 plays in functional recovery of neural pathways should be further elucidated.

Several transcriptional changes in the DRG occur because of intramuscular delivery of AAV1-CMV-NT3 in rats with pyramidotomy (Kathe and Moon, 2018). These include upregulation of *Sema4c*, which belongs to a family of axon guidance molecules and cytoskeletal remodelling associated genes like *Gas7.* Such transcriptional changes could stabilise afferent contacts on motor neurons, strengthen proprioceptive circuitry and be responsible for reported improved skilled motor function (Kathe et al., 2016). NT3 treatment reduced afferent input onto *gastrocnemius* α-motor neurons in our study, suggesting recovery of precision ladder stepping may require, in part, normalisation of vGlut1+ input. Our previous work has shown that despite elevated circulatory NT3 following treatment, spinal reflexes, and the number of vGlut1+ boutons only normalised on circuits where the afferent nerve came froma treated muscle (Kathe et al., 2016). As such, we would expect the normalisation of vGlut1+ input in this study to be specific to afferent connections of the injected muscles. Of note is the increase we show in corrective steps. This may be explained by more effective proprioceptive feedback, enabling detection of a slip early and repositioning of the paw before transfer of weight support and slippage. As slipping would involve activation of Ia afferents innervating flexors and extensors of the ankle joints, it is possible that proprioceptive circuitry involving the ankle flexors and extensors was abnormal after injury. However, note that in our study the H-reflex was recorded instead from an intrinsic hindpaw muscle.

### 4.3 T9 contusion model did not reliably induce spasms or hyperreflexia

Our study reveals that hindlimb spasms are not reliably generated by a 250kDyn contusion at the T9 spinal level in female Wistar rats, contrasting with previous reports using the same severe contusion injury model (Ryu et al., 2017; Chang et al., 2019) or a T-lesion at the same level (Gonzenbach et al., 2010). Others have demonstrated plantar hindlimb muscle hyperreflexia and visible hindlimb spasms during swimming, walking and in their home cage using female Sprague Dawley rats using this severe contusion injury model (Ryu et al., 2017; Ryu et al., 2021). Reasons for the differences could be slight alterations in equipment, methodologies employed, or strains used (Mills et al., 2001). Our injuries were not as functionally extensive as those previously reported with this injury (Chang et al., 2019), suggesting the CST was not as comprehensively injured, which may account for the lack of visible spasms (Gonzenbach et al., 2010). Alternatively, spared reticulospinal function is positively correlated with the presence of hindlimb spasticity in SCI patients (Sangari and Perez, 2019), suggesting we may have excessively damaged this pathway. It is possible that altering the methods employed may have yielded different data. For example, EMG recordings from affected muscles may detect mild spasms, or a train of stimuli may fully depress the H reflex (Lee et al., 2014) compared to paired pulses (Chang et al., 2019; Ryu et al., 2017). However, as the methods employed have been successful in several prior studies, this study highlights that production of visually identifiable spasms and hyperreflexia of the hindpaw is not as a reliable outcome of severe T9 contusive injuries as previously reported.

## 5 Conclusion

We show that targeted, delayed delivery of NT3 to hindlimb and trunk muscles 48 h following a severe contusive injury to the thoracic spinal cord improved trunk stability, accuracy of stepping during skilled locomotion and alternation of the hindlimbs during swimming but had no effect on gross locomotor function in the open field. We suggest that this increase was through modulated proprioceptive feedback, as vGlut1+ boutons on presumptive proprioceptive afferents innervating treated muscles were normalised. Further, here we show that a T9 250kDyn contusion does not reliably result in measurable signs of spasticity, such as hindlimb spasms and hyperreflexia. This is the first demonstration that a clinically feasible 48-h delay in AAV1-NT3 treatment application can help restore precise motor control in functionally compromised hindlimbs, supporting its use as a therapeutic strategy for SCI and continued clinical development.

### Competing interests

The authors report no financial or non-financial competing interests associated with this work. The experiments were all completed before L.D.F.M. moved to Spark Therapeutics.

### Author contributions

Animal work, data acquisition, tissue processing, immunohistochemistry, imaging, and data analysis were performed by J.D.S-S. and A.K., manuscript preparation was performed by P.M.W and L.D.F.M., and manuscript editing was performed by all the authors. The project was conceived and designed by P.M.W and L.D.F.M. in consultation with A.K. and J.D.S-S. All authors have approved the final version of the manuscript, agree to be accountable for the work, and qualify of authorship.

### Financial support

MRC project grant (MR/S011110/1) and King’s College London Prize Fellowship to P.M.W; and a Nathalie Rose Barr PhD studentship from the Spinal Research Trust (NRB117 to LDFM for JS-S).

## Data Availability

Data is available through odc-sci.org

## Appendix A Supplementary data

Supplementary material Image 1
