# Supplementary material for: Delayed viral vector mediated delivery of neurotrophin-3 improves skilled hindlimb function and stability after thoracic contusion
Source: Exp Neurol. 2023 Feb;360:114278. doi: 10.1016/j.expneurol.2022.114278 (PMC10227192; doi:10.1016/j.expneurol.2022.114278)
Supplement: Supplementary file 1 — Supplementary material [file mmc1.docx]

**Delayed viral vector mediated delivery of neurotrophin-3 improves skilled hindlimb function and stability after thoracic contusion in rats**

**Authors:** Jared D. Sydney-Smith^1,^*, Alice M. Koltchev^1,2,^*, Lawrence D. F. Moon^1,3,§^, Philippa M. Warren^1,§^

**Affiliations:**

^1^ The Wolfson Centre for Age-Related Diseases, Guy's Campus, King's College London, London Bridge, London SE1 1UL, UK

^2^ Now at: Sainsbury Wellcome Centre for Neural Circuits and Behaviour, University College London, London W1T 4JG, UK.

^3^ Now at: Spark Therapeutics, 3025 Market Street, Philadelphia, PA, 19104, USA.

* joint first authors

§ joint senior authors

**Supplementary Figures 1-3:**

Supplementary Figure 1: Thoracic contusion injury causes bilateral effects on hindlimb function.

Supplementary Figure 2: Thoracic contusion permanently increases hindlimb miss-stepping in skilled tasks

Supplementary Figure 3: H reflex parameters did not alter in unexcluded animals following thoracic contusion.

**Supplementary Table 1:**

Supplementary Table 1: Recovery of hindlimb performance on the horizontal ladder varied by limb for "misses” and “corrective steps” and a trend to a difference for “slips”.


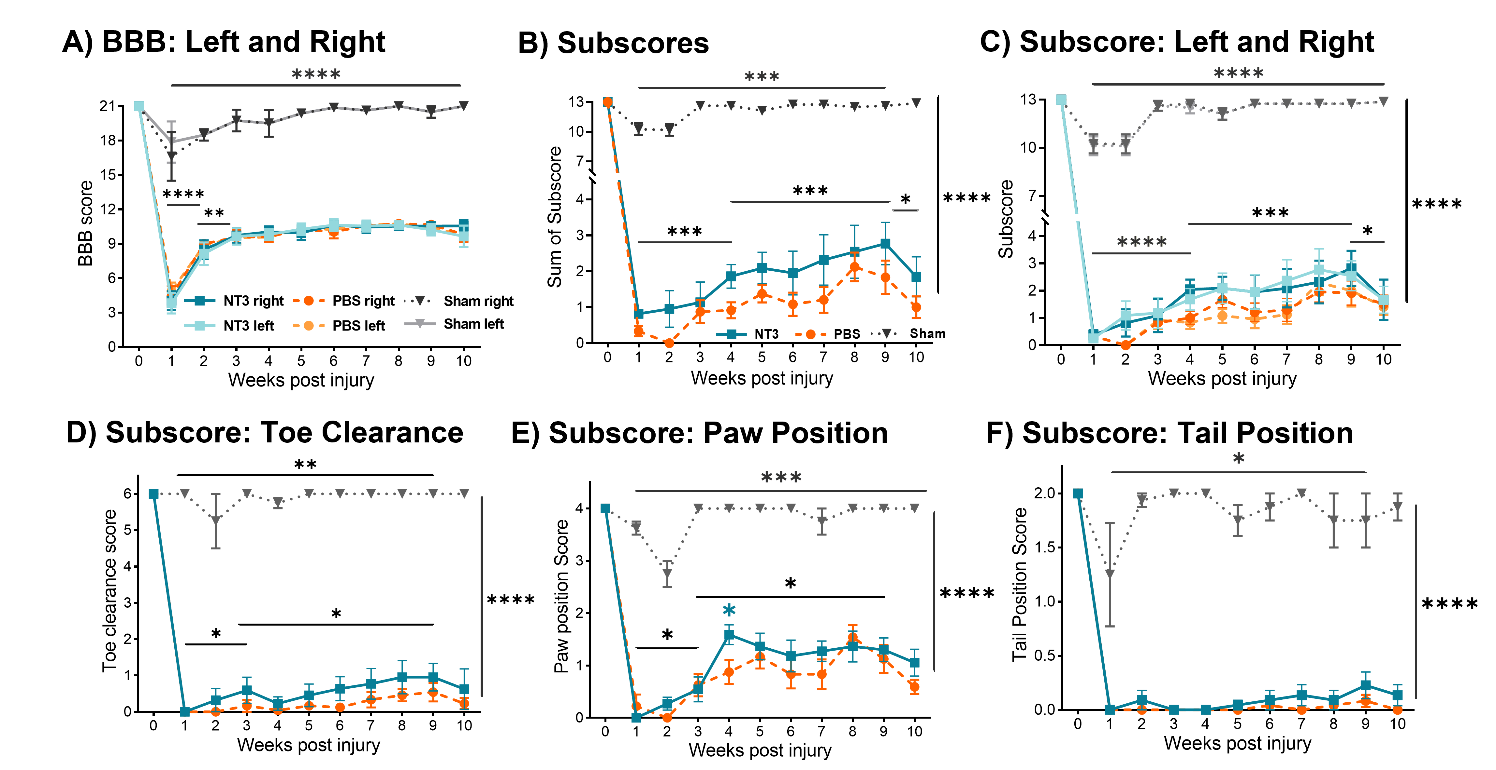
**Supplementary Figure 1:** Thoracic contusion injury causes bilateral effects on hindlimb function. A) BBB scores showed no functional difference between the injury induced deficits of the left and right hindlimbs of either the NT3 or control groups, indicating that the contusion was functionally bilateral. Locomotion gradually improved from weeks 1 to 3 post injury in the injured cohorts, occuring similarly in both the left and right hindlimb, Left = light colours, right = dark colours. B) BBB subscores were reduced after injury but did improve over time in both injured groups from weeks 1 to 3. Overall, locomotion was not improved by NT3 treatment compared to controls. C) Deficits in BBB subscore were comparable between left and right hindlimbs in all injured animals, with a similar recovery. Injured animals made a similar recovery over time in both hindlimbs. Legend as in panel A. D-F) Component sections of the BBB were analysed showing D) average toe clearance, which improved in both NT3 and control animals over time, with the NT3 group having a trend towards higher average toe clearance. No evidence for an effect of NT3 was detected. E) Average paw position was imperfectly placed after injury with some recovery occurring in both NT3 and control animals to 9 weeks. NT3 treatment caused improvement in paw positioning at 4 weeks but this was not maintained. F) Tail positioning during locomotion was altered after injury. However, NT3 did not cause a recovery in this specific function.


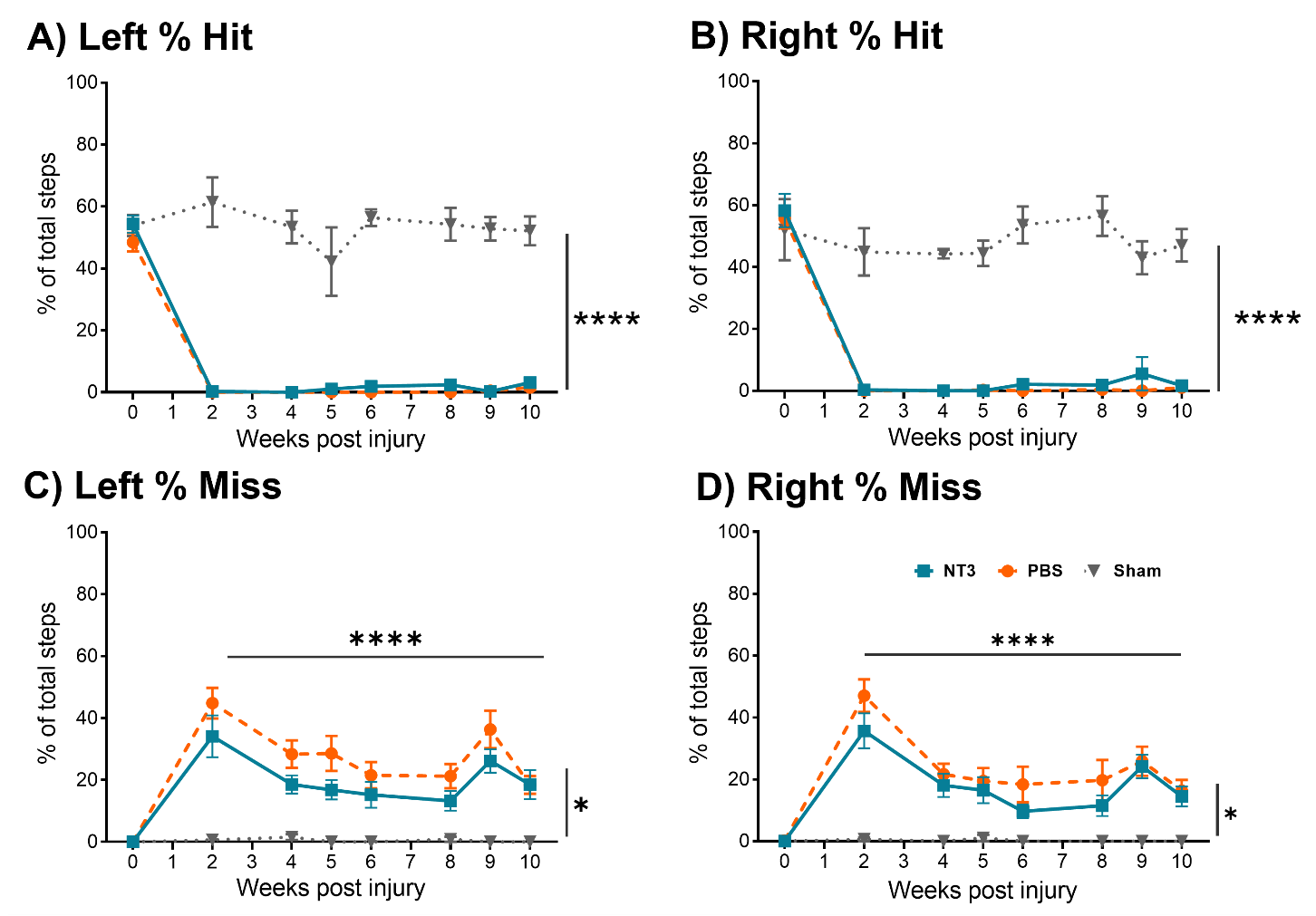


Supplementary Figure 2: Thoracic contusion permanently increases hindlimb mis-stepping in skilled tasks. The thoracic contusion caused a permanent decrease in the number of error-free steps by the A) left and B) right hindlimbs with no increase shown in the NT3 group compared to injured control. C-D) Further, the contusion injury caused a substantive increase in the number of bilateral miss-steps performed by all animals which was similarly not recovered following NT3 treatment. Baseline recordings were not used as a covariate but were excluded for tests of effects and interactions. For NT3 n=11 for PBS n=11, for sham n=4.


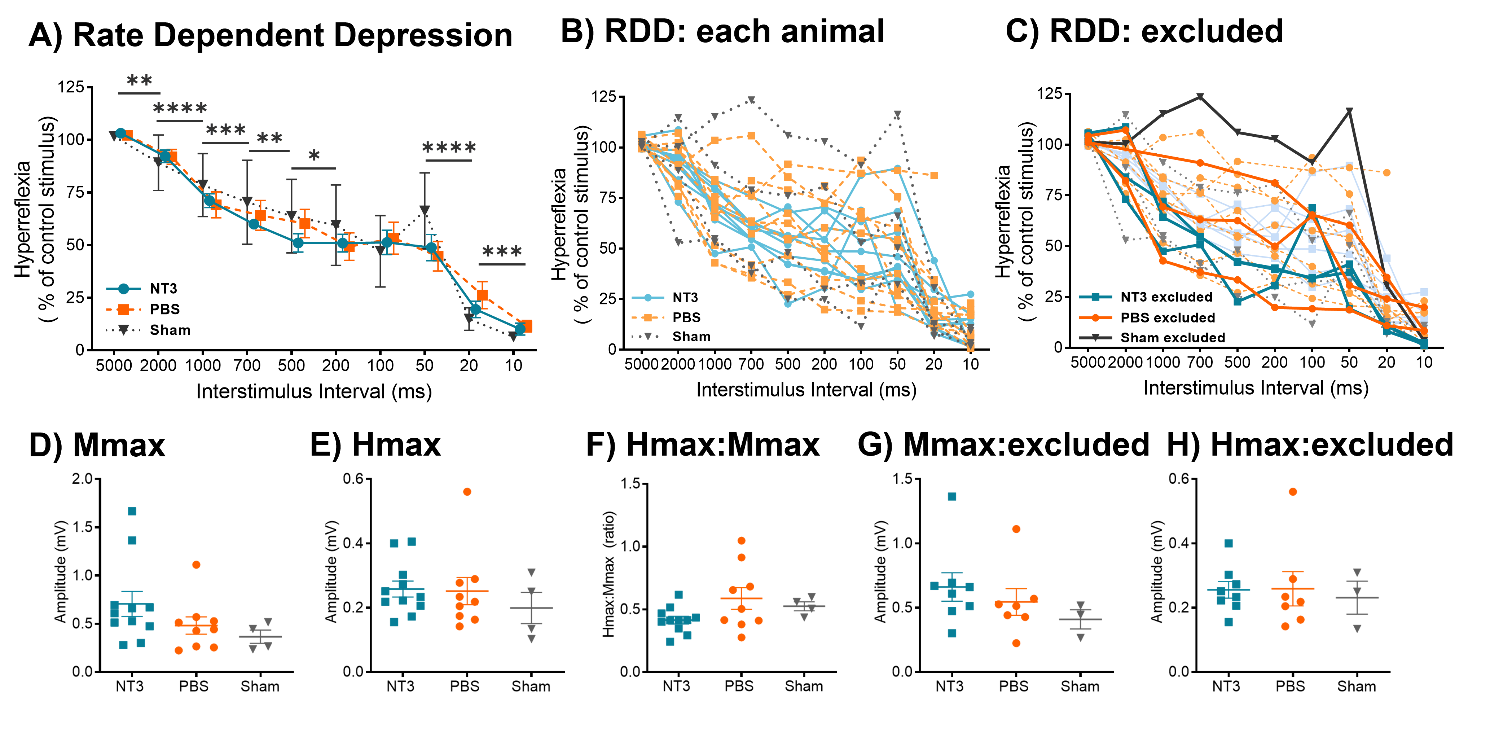


**Supplementary Figure 3:** H reflex parameters did not alter in unexcluded animals following thoracic contusion. A) H wave of all animals showing RDD was present in comparable amounts in both injured and sham animals (effect of group, linear model, F(2,22)=0.14, P=0.87), with no treatment interaction present (effect of group x interstimulus interval, linear model, F(18,185)=1.09, P=0.42). B) RDD for every animal prior to exclusion. C) RDD of animals excluded from analysis showing similar trends to the group data. D) The maximum amplitude of the M wave was comparable between all contused and sham animals (one way ANOVA, F(2,21)=1.86, p=0.18). E) The maximum amplitude of the H wave was comparable between all contused and sham animals (one way ANOVA, F(2,21)=0.50, p=0. 61). F) The Hmax:Mmax ratio, an indicator of reflex excitability, was comparable between all animals of the injured and sham groups (One way ANOVA, F(2,21)=2.41, p=0.11). G) The maximum amplitude of the M wave was comparable between contused and sham animals following exclusions (one way ANOVA, F(2,15)=0.94, p=0.41). H) The maximum amplitude of the H wave was comparable between contused and sham animals following exclusions (one way ANOVA, F(2,15)=0.076, p=0.93).

| **Dependent variable** | **Effect of side** | **Effect of group** | **Effect of time** | **Interaction of group x time** | **Effect of baseline** | **Post hoc NT3 v PBS** | **Post hoc NT3 v Sham** | **Post hoc PBS v Sham** |
| --- | --- | --- | --- | --- | --- | --- | --- | --- |
| Slips | 0.087 | <0.001 | <0.001 | 0.025 | <0.001 | 0.002 | <0.001 | <0.001 |
| Hits | 0.101 | <0.001 | <0.001 | <0.001 | 0.897 | 0.314 | <0.001 | <0.001 |
| Misses | 0.006 | 0.002 | <0.001 | <0.001 | n/a | 0.139 | 0.009 | 0.139 |
| Corrective steps | 0.004 | <0.001 | <0.001 | 0.001 | 0.023 | 0.010 | 0.046 | <0.001 |

**Supplementary Table 1:** Recovery of hindlimb performance on the horizontal ladder varied by limb for "misses” and “corrective steps”, with a trend to a difference for “slips”. Linear models were run for each dependent variable separately with the following factors included: side, group, time, group x time, baseline. Fit of the model was assessed for each of three covariance structures, selecting the one with the lowest value for Akaike’s Information Criterion as described previously (Duricki et al., 2016). Post hoc tests (LSD) are provided for the factor “group”. Values denote p values. n/a denotes baseline omitted from model since no animal made any misses during baseline testing.

**References:**

DURICKI, D. A., HUTSON, T. H., KATHE, C., SOLEMAN, S., GONZALEZ-CARTER, D., PETRUSKA, J. C., SHINE, H. D., CHEN, Q., WOOD, T. C., BERNANOS, M., CASH, D., WILLIAMS, S. C. R., GAGE, F. H. & MOON, L. D. F. 2016. Delayed intramuscular human neurotrophin-3 improves recovery in adult and elderly rats after stroke. *Brain,* 139**,** 259-275.
